# Treatment of penetrating injuries to the retrohepatic inferior vena cava: a systematic review

**DOI:** 10.1590/1677-5449.202401492

**Published:** 2026-02-06

**Authors:** Adenauer Marinho de Oliveira Góes, Simone de Campos Vieira Abib, Gustavo Henrique Dumont Kleinsorge, Daniella Adrea Araujo Rossi Vieira, Luis Carlos Uta Nakano, Mariseth Carvalho de Andrade

**Affiliations:** 1 Universidade Federal do Pará – UFPA, Belém, PA, Brasil.; 2 Centro Universitário do Estado do Pará – CESUPA, Belém, PA, Brasil.; 3 Universidade Federal de São Paulo – UNIFESP-EPM, São Paulo, SP, Brasil.; 4 Fundação Hospitalar do Estado de Minas Gerais, Hospital João XXIII, Clínica de Cirurgia Vascular, Belo Horizonte, MG, Brasil.; 5 Centro Universitário Metropolitano da Amazônia – UNIFAMAZ, Belém, PA, Brasil; 6 Fundação Santa Casa de Misericórdia do Pará – FSCMPA, Belém, PA, Brasil.

**Keywords:** inferior vena cava, wounds and injuries, endovascular procedures, therapeutics, surgical procedures, operative, systematic review

## Abstract

**Background:**

Injuries to the retrohepatic vena cava are associated with high mortality rates and vascular control must be obtained prior to exposure. Various treatment techniques have been described, including triple hepatic vascular exclusion, atriocaval shunt, and endovascular and hybrid strategies.

**Objectives:**

To determine which of these is associated with the lowest mortality rate.

**Methods:**

A systematic literature review was conducted, guided by the Cochrane Handbook and PRISMA guidelines. The PUBMED, LILACS, Embase, Web of science, and Scopus databases were searched and Ryyan software was employed to manage the studies identified.

**Results:**

Sixteen studies were selected, reporting 96 cases, in 49 of which the patients were treated with triple hepatic exclusion, in 38 with an atriocaval shunt, and in 9 with endovascular or hybrid techniques, with the third of these groups being statistically less frequent (p < 0.0001). The mortality rate was 53.8%, with no statistically significant differences between any of the techniques studied (p = 0.9085).

**Conclusions:**

Injuries to the retrohepatic vena cava had similar mortality rates regardless of the technique employed for treatment.

## INTRODUCTION

The inferior vena cava (IVC) is the vessel most frequently involved in penetrating abdominal traumas.^1,2^ One of the factors with a direct influence on mortality is the anatomic segment that is injured, with rates that can be as high as 63.3% when the infrarenal segment is involved and may reach 100.0% in retrohepatic injuries.^3-6^

Operative exposure of IVC injuries without obtaining prior vascular control can cause massive bleeding and death. However, specific injuries to the retrohepatic segment demand complex surgical maneuvers, both to obtain vascular control and to expose the injury itself.^7-11^

Over the years, two techniques have become consolidated for obtaining vascular control of injuries to the retrohepatic vena cava: triple hepatic exclusion (THEx), also known as total vascular isolation of the liver and first described by Heaney in 1966, and the atriocaval shunt (ACS), originally described in 1968 by Schrock.^12-14^

The advantage of an ACS is maintenance of venous return via the IVC axis, ameliorating the reduction of cardiac output caused by clamping above the renal veins, which is a necessary part of the THEx maneuver. On the other hand, vascular isolation of the liver does not require a cardiac chamber to be opened, avoiding a series of complications that can be related to an ACS.^15-17^

Since 1998, there have been a growing number of reports in the literature of endovascular techniques, such as placement of covered stents, and of hybrid strategies, such as endovascular balloon occlusion for temporary hemostasis, followed by surgical management of the injury. These techniques constitute less invasive techniques and may be associated with improved outcomes. However, the need for availability of materials and a trained team for this type of procedure limits their use.^18-22^

While there is consensus among researchers on the elevated lethality associated with traumas to the retrohepatic segment of the IVC, the literature does not provide a consistent definition of which strategy for treating these injuries yields the lowest mortality.^6,11,23^

## OBJECTIVE

To define which technique for treatment of penetrating injuries to the retrohepatic segment of the IVC yields the lowest mortality and incidence of complications in adult patients treated with emergency surgery.

## METHODS

This systematic literature review was registered on the PROSPERO platform,^24^ under registration number CRD42023464133, and the study protocol has been published previously.^25^ The study is exempt from submission for Ethics Committee approval because it is a systematic review of the literature.

### Eligibility criteria

#### Types of studies

The review process was developed using the Cochrane Handbook for Systematic Reviews of Interventions^26^ and the Preferred Reporting Items for Systematic Reviews and Meta-Analyses (PRISMA) 2020. Randomized controlled clinical trials (RCTs) with parallel or cluster designs and quasi-RCTs were included. Non-randomized intervention studies were also considered for inclusion providing they studied at least two comparative groups of interest, as were observational studies, cases series, and case reports if no RCTs or quasi-RCTs could be found that covered a subject of interest.

All studies that described cases of penetrating injuries to the retrohepatic segment of the IVC in adult patients treated in an emergency surgery scenario with THEx, ACS, or endovascular/hybrid (E/H) techniques were classified.

#### Types of participants

Patients of both sexes aged 18 years or over were included if they had undergone emergency surgery for penetrating injuries to the retrohepatic segment of the IVC, confirmed by imaging exams or surgical exploration.

#### Types of intervention

The following types of intervention for treatment of penetrating injuries to the retrohepatic segment of the IVC were included in the review: THEx, ACS, and E/H techniques.

### Sources of information

#### Search method used to identify studies

A search string was constructed using Medical Subject Headings keywords (((vena cava[Title/Abstract]) AND (trauma OR injuries)[Title/Abstract])) and used to search the following databases: Literatura Latino-Americana e do Caribe em Ciências da Saúde, Web of Science, PubMed/MEDLINE, Scopus, and Embase. No results filters were set. A search for prior reviews covering the same subject was run on the Cochrane Library system during the planning phase, which did not detect any relevant publications. Ongoing and unpublished trials were also sought on ClinicalTrials.gov and the International Clinical Trials Registry Platform, via the World Health Organization (WHO) portal.

#### Selection of studies

Study selection comprised three stages. In stage one, Rayyan^27^ software was used to manage the articles identified by the database searches, classifying each item and excluding duplicates manually. In stage two, two reviewers independently analyzed the titles and abstracts of the selected articles, applying the predefined eligibility criteria. The third stage was conducted by two different reviewers, who independently read the texts of each article selected in the previous stage, selecting the final list of studies used for data extraction.

The selection process is illustrated in a PRISMA 2020 flowchart.^28^ Any differences of opinion that emerged during this process were resolved by discussion between the study team members.

#### Data extraction and management

The data extraction form (Table 1) was completed by two reviewers independently, extracting the following variables from the articles:

**Table 1:** Data collection form.

**Variables**	**Result**
Article code:	
Authors:	
Title:	
Year of publication:	
Country:	
Study classification:	
Study design:	
Method of analysis:	
Jornal:	
Total sample size:	
Total number of retrohepatic cava injuries:	
Number of participants followed-up:	
ACS:	
THEx:	
E/H techniques:	
Mean age:	
Minimum age:	
Maximum age:	
Males:	
% male:	
Females:	
% female:	
Mean duration of follow-up:	
Minimum duration of follow-up:	
Maximum duration of follow-up:	
ACS mortality rate (%):	
THEx mortality rate (%):	
E/H mortality rate (%):	
Overall mortality rate (%):	
Blood products, ACS (ml):	
Blood products, THEx (ml):	
Blood products, E/H (ml):	
Blood products, overall (ml):	
Mean ICU stay, ACS:	
Minimum ICU stay, ACS:	
Maximum ICU stay, ACS:	
Mean ICU stay, THEx:	
Minimum ICU stay, THEx:	
Maximum ICU stay, THEx:	
Mean ICU stay, E/H:	
Minimum ICU stay, E/H:	
Maximum ICU stay, E/H:	
Overall mean ICU stay:	
Overall minimum ICU stay:	
Overall maximum ICU stay:	
Patients needing hemodialysis, THEx:	
Patients needing hemodialysis, E/H:	
Type of population (penetrating or blunt trauma):	
Risk of bias:	
What risk of bias:	

ACS = atriocaval shunt; THEx = triple hepatic exclusion; E/H = endovascular/hybrid; ICU = intensive care unit.

- Study design;
- Method of analysis;
- Outcome measures;
- Duration of follow-up;
- Number of participants at baseline and follow-up;
- Type of population;
- Percentage (%) by sex;
- Mean age (standard deviation [SD]);
- Adjusted covariates;
- Interventions used;
- Data used to calculate differences in clinical outcomes between results of interventions: percentage survival, use of blood products, time spent in intensive care unit, need for hemodialysis;
- Sources of funding for the study and authors’ declarations of conflicts of interests;

#### Statistical analysis

Information on sample characteristics was input to a Microsoft® Office Excel® 2016 spreadsheet. Descriptive statistics were presented in tables and used to plot graphs.

For statistical analysis, goodness-of-fit tests were used for univariate tables, the chi-square test of independence was used for bivariate comparisons, and one-criterion variance analysis was used to compare mortality rates between different techniques.

Descriptive and analytical analyses were conducted using BioEstat 5.4, with a significance level of α = 0.05, or 5%.

## RESULTS

**Figure 1:**
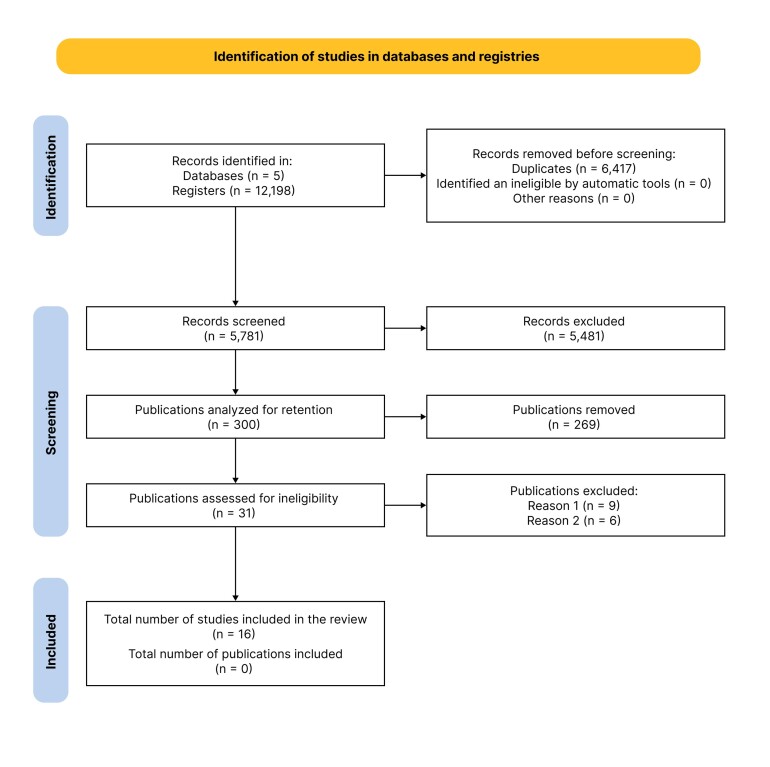
PRISMA 2020 flow diagram. PRISMA = Preferred Reporting Items for Systematic reviews and Meta-Analyses. Reason 1 = articles did not meet inclusion criteria; Reason 2 = inadequate samples.

The search strategy located 12,198 studies, 6,417 (52%) of which were duplicates. Screening of titles and abstracts excluded 5,481 articles, the majority because they described traumatisms involving other segments of the IVC rather than the retrohepatic portion. Analysis of the full texts resulted in exclusion of a further 284 articles that did not meet the inclusion criteria. As a result, a total of 16 articles were included in the review. Figure 1 shows the PRISMA flow diagram illustrating the study selection process.

The selected studies were published from 1970 to 2021, with more than 50.0% published between 1970 and 1999 (Table 2). Cases series made up 81.3% of the sample, while case reports accounted for a further 18.7%. The searches did not locate any parallel RCTs, cluster RCTs, or quasi-RCTs. Table 2 summarizes the selected articles.

**Table 2:** Articles selected for the review.

**Title**	**Authors**	**Year of publication**	**Country**
Successful Management of an Injury to the Suprarenal Inferior Vena Cava^29^	Bricker DL, Wukasch DC.	1970	United States
Massive Venous Injuries Associated With Penetrating Wounds Of The Liver^30^	Burns RP, Britt LG.	1975	United States
Traumatic injuries of the inferior vena cava^31^	Graham JM, Mattox KL, Beall AC, DeBakey ME.	1978	United States
Inferior vena cava injuries - a continuing challenge^32^	Millikan JS, Moore EE, Cogbill TH, Kashuk JL.	1983	United States
Abdominal Venous Injuries^33^	Wiencek RG, Wilson RF.	1986	United States
Contemporary management strategy for major inferior vena caval injuries^34^	Klein SR, Baumgartner FJ, Bongard FS.	1994	United States
Penetrating injuries of the abdominal inferior vena cava^35^	Deagiannis E, Velmahos G C, Levy R D, Souter I, Benn C A, Saadia R.	1996	Africa
The ongoing challenge of retroperitoneal vascular injuries^36^	Coimbra R, Hoyt D, Winchell R, Simons R, Fortlage D, Garcia J.	1996	United States
Management of penetrating juxtahepatic inferior vena cava injuries under total vascular occlusion^37^	S C Khaneja, W F Pizzi, P S Barie, N Ahmed.	1997	United States
Prognostic factors in patients with inferior vena cava injuries ^38^	Rosengart MR, Smith DR, Melton SM, May AK, Rue LW.	1999	United States
Abdominal vena caval injuries: Outcomes remain dismal^39^	Hansen CJ, Bernadas C, West MA, Ney AL, Muehlstedt S, Cohen M, Rodriguez JL.	2000	United States
Repair of a juxta hepatic inferior vena cava injury using a simple endovascular technique^40^	Angeles AP, Agarwal N, Lynd C Jr.	2004	United States
Shunt atriocava. Report of two cases^41^	Soto S, Oettinger R.	2005	Chile
Traumatismos de veia cava inferior^7^	Costa CA, Baptista-Silva JCC, Rodrigues LME, Mendonça FLP, Paiva TS, Burihan E.	2005	Brazil
Outcome of ligation of the inferior vena cava in the modern era^42^	Sullivan PS, Dente CJ, Patel S, Carmichael M, Srinivasan JK, Wyrzykowski AD, Nicholas JM, Salomone JP, Ingram WL, Vercruysse GA, Rozycki GS, Feliciano DV.	2010	United States
Lessons learned from treating 114 inferior vena cava injuries at a limited resources environment^6^	Góes Junior AMO, Silva KTB, Furlaneto IP, Abib SCV.	2021	Brazil

Overall, the selected articles described 921 cases of IVC injury, although only 206 (22.4%) involved the retrohepatic segment. Of these, follow-up was described for 195 patients (21.2%).

Male patients accounted for 90.3% of the study population, while 9.7% of the patients were female, and mean age was 27.3 years.

It was possible to identify the treatment technique used in 96 cases. There was no statistically significant difference in the frequency of use of THEx (49 cases) and ACS (38 cases). However, use of E/H techniques was statistically less frequent (nine cases) (p < 0.0001).

Overall mortality of cases with retrohepatic IVC injuries was 53.8%. Cases treated with THEx had 43.0% mortality; cases with ACS implantation had 46.4% mortality; and E/H techniques were associated with 34.0% mortality. However, comparison of the mortality rates associated with each of the three techniques did not detect any statistically significant difference (p = 0.9085). Table 3 lists the statistical data on the mortality comparisons.

**Table 3:** Statistical analysis by ANOVA for comparison of overall and between-techniques mortality.

**Variables**	**Number of cases**	**Mortality rate**		**p value** *
		**Variance**	**Mean**	
Overall mortality	96	0-93%	53.8%	
ACS technique	38	0-100%	46.4%	0.9085
THEx technique	49	0-80%	43.0%	
E/H technique	9	0-68%	34.0%	
ACS x THEx	----	----	----	0.2723
ACS x E/H	----	----	----	0.2351
THEx x E/H	----	----	----	0.3706
p value	< 0.0001	----	-----	----

ACS = atriocaval shunt; THEx = triple hepatic exclusion; E/H: endovascular/hybrid; ANOVA = analysis of variance.

* p value 0.

With regard to the variables needed for comparisons of clinical outcomes, information on use of blood products, length of stay in the intensive care unit, and need for hemodialysis were not reported in the majority of articles, preventing analysis of these variables.

## DISCUSSION

Improvements in pre-hospital care and reduction of the time taken to transport patients have enabled severely traumatized patients, who in the past would have died at the scene, to reach a hospital alive. Such cases demand rapid and effective management.^30,34,43-45^

The elevated mortality associated with retrohepatic IVC injuries is related to the technical difficulty of surgical access, the velocity of blood volume loss, the size of the injury, and the time elapsed before hemostasis is achieved.^35,38-40,46,47^ The overall mortality rate observed in this study was greater than 50%, in line with what is described in literature on the subject, illustrating the challenge that this type of trauma presents.^31,33,42^

The literature presents conflicting data on ACS, as described by Schrock in 1968. In addition to the elevated mortality, an ACS demands complex surgical maneuvers, such as insertion of the shunt via an opening in the right atrium, with purse string suture. In addition to the technical difficulty of the method, there are reports of occurrence of air embolism and cardiac and vascular injuries during placement of the shunt.^32,48-50^ However, there are also successful reports and adherents of the technique claim that maintaining venous return via the inferior vena cava axis while injuries are repaired reduces the hemodynamic repercussions of hypovolemia, offering advantages with respect to occlusion of the vena cava, which is necessary to perform THEx.^41^

In turn, THEx is also associated with elevated mortality. However, the absence of any statistically significant difference in mortality in relation to ACS suggests that the elevated lethality is not associated with the strategy chosen for treatment (p = 0.2723).

While THEx can be achieved clamping the vena cava between the liver and the diaphragm, this option should be reserved for elective procedures, such as liver transplantation,^12^ because the minimal space available increases the risk of iatrogeny. In the context of trauma, when access must be obtained rapidly, the safest method for obtaining control of the inferior cava above the liver is at the intrapericardial portion, which can be exposed by sternotomy with a right anterolateral thoracotomy,^51^ which tends to be preferred by authors, since the communication between the two accesses (thoracic and abdominal), with which a thoraco-phreno-laparotomy is also possible, enables considerable hepatic mobility, facilitating exposure of the vascular injury.^52^

The reduction in venous return caused by occlusion of the inferior vena cava can reduce arterial blood pressure. However, clamping the descending aorta via a left anterolateral thoracotomy or the supraceliac segment of the abdominal aorta may partially compensate for the hemodynamic effects of venous occlusion in patients with systolic arterial blood pressure less than 90 mmHg.^37^

The case series identified for this systematic review reported mortality from 30 to 55% in patients treated with THEx,^35,36^ and from 68 to 100%^29,41,42^ when ACS was employed. However, since no statistically significant difference in mortality was detected between the two strategies, it falls to the surgeon to decide which technique to use, considering their training and experience and the availability of resources.

The low number of cases in which E/H techniques were used was expected, not only because these are options that have been developed more recently, but also because of the scarcity of the technical resources and material needed to perform them. In view of the small number of cases described in the articles selected this review, cases treated exclusively with endovascular techniques and those treated using hybrid strategies were analyzed together as a single group.

Although the Resuscitative Endovascular Balloon Occlusion of the Aorta technique was originally described for aortic occlusion with an endovascular balloon, it has come to be used for treatment of IVC injury as well, leading to coining of the term Resuscitative Endovascular Balloon Occlusion of the Vena Cava. From the 2000s onwards, descriptions of use of covered stents for vena cava trauma have become ever more frequent.^53-57^

Exclusively endovascular techniques, such as placement of covered stents, and also hybrid strategies, offer minimally invasive and effective options for treatment of complex traumas.^58-60^ According to the present literature review, they are associated with relatively low mortality rates and should undoubtedly be considered when available.

Hybrid strategies involve obtaining vascular occlusion by inflating intraluminal balloons, inserted via percutaneous access or by surgical exposure of vessels. This approach can be used to achieve temporary hemostasis, providing a relatively bloodless operating area, facilitating vascular repairs with a lower risk of significant stenosis, and reducing the likelihood of occlusion of tributaries of the vena cava.^61-63^

Although the mortality rates associated with THEx, with ACS, and with E/H techniques were not statistically different in this review, this result could be related to the limited sample size and to the heterogeneous nature of the groups being compared.

Limitations of this review include the fact that higher quality studies were not found, only case reports and cases series were included, which exhibited considerable heterogeneity in terms of the description of variables. All of the studies also described related injuries to other anatomic structures and none of them were designed to compare the outcomes of the three interventions analyzed. Moreover, out of four variables listed in our review protocol^25^ to be used to assess the clinical outcomes of interventions (percentage survival, use of blood products, length of stay in intensive care, and need for hemodialysis), only patient survival was reported in all of the selected articles, introducing a bias that compromised the statistical analysis and had a negative impact on evidence level. Moreover, in all cases there were other injuries involved, which precludes the conclusion that the outcomes described are specifically attributable to injury to the retrohepatic segment of the IVC, introducing biases due to lack of data and presence of confounding factors.

The protocol for this systematic review^25^ envisaged use of the ROBINS-I instrument to assess risk of bias in the selected studies, since a preliminary survey had only identified non-randomized studies of this subject. However, the absence of cohort and case-control studies comparing the three types of intervention investigated, compounded by the limitations of the included studies, meant that the instrument proposed in the initial protocol could not be used.

Multicenter prospective studies with standardized collection of data on progression and outcome of cases and on the techniques employed could contribute to attenuating the limitations described and to more precise conclusions. However, the severity of the type of injury studied makes it difficult to achieve homogeneous conduct, since the expertise and material needed are not uniformly available, meaning that it is unlikely that studies of this nature will be conducted.

## CONCLUSIONS

This review of the outcomes of cases of penetrating injuries to the retrohepatic segment of the IVC in adult patients treated in emergency scenarios using ACS, THEx, or E/H techniques did not demonstrate either superiority or inferiority of any the techniques in terms of mortality.

## Data Availability

Os dados que sustentam este estudo estão disponíveis mediante solicitação ao autor correspondente, AMOG (goesjunior@ufpa.br).
