# Supplementary material for: Treatment of penetrating injuries to the retrohepatic inferior vena cava: a systematic review
Source: J Vasc Bras. 2026 Feb 6;24:e20240149. doi: 10.1590/1677-5449.202401492 (PMC12974593; doi:10.1590/1677-5449.202401492)
Supplement: Translation

# Translation: Tratamento de lesões penetrantes de veia cava retro-hepática: uma revisão sistemática

**DOI:** 10.1590/1677-5449.202401491

**Authors:** Adenauer Marinho de Oliveira Góes, Simone de Campos Vieira Abib, Gustavo Henrique Dumont Kleinsorge, Daniella Adrea Araujo Rossi Vieira, Luis Carlos Uta Nakano, Mariseth Carvalho de Andrade

**Affiliations:** 1 Universidade Federal do Pará – UFPA, Belém, PA, Brasil.; 2 Centro Universitário do Estado do Pará – CESUPA, Belém, PA, Brasil.; 3 Universidade Federal de São Paulo – UNIFESP-EPM, São Paulo, SP, Brasil.; 4 Fundação Hospitalar do Estado de Minas Gerais, Hospital João XXIII, Clínica de Cirurgia Vascular, Belo Horizonte, MG, Brasil.; 5 Centro Universitário Metropolitano da Amazônia – UNIFAMAZ, Belém, PA, Brasil; 6 Fundação Santa Casa de Misericórdia do Pará – FSCMPA, Belém, PA, Brasil.

**Keywords:** veia cava inferior, ferimentos e lesões, procedimentos endovasculares, procedimentos de tratamento, procedimentos cirúrgicos operatórios, revisão sistemática

## Abstract

**Contexto:**

As lesões da veia cava retro-hepática apresentam alta mortalidade e requerem controle vascular prévio à sua exposição. Para seu tratamento, foram descritas técnicas como a tríplice exclusão hepática, o *shunt* átrio-caval e técnicas endovasculares ou híbridas.

**Objetivos:**

Definir qual dessas técnicas apresenta menor mortalidade.

**Métodos:**

Foi realizada uma revisão sistemática da literatura, orientada pelo *Cochrane Handbook* e pela declaração Preferred Reporting Items for Systematic Reviews and Meta-Analyses. As bases de dados PubMed, Literatura Latino-Americana e do Caribe em Ciências da Saúde, Embase, Web of Science e Scopus foram pesquisadas, e o *software* Rayyan foi utilizado para o gerenciamento dos estudos.

**Resultados:**

Ao todo, 16 estudos foram selecionados, resultando em 96 casos: 49 pacientes foram tratados com tríplice exclusão hepática, 38 com *shunt* átrio-caval e 9 com técnicas endovasculares ou híbridas, sendo estas últimas significativamente menos frequentes na amostra (p < 0,0001). A mortalidade global foi de 53,8%, sem diferença estatisticamente significativa entre as técnicas analisadas (p = 0,9085).

**Conclusões:**

As lesões da veia cava retro-hepática apresentaram mortalidade semelhante, independentemente da técnica utilizada para o tratamento.

## INTRODUÇÃO

A veia cava inferior (VCI) é o vaso mais frequentemente acometido nos traumatismos abdominais penetrantes^1,2^. Um dos fatores que influencia diretamente a mortalidade é o segmento anatômico lesionado, podendo atingir 63,3% em sua porção infrarrenal e até 100,0% no segmento retro-hepático^3-6^.

A exposição das lesões da VCI sem controle vascular prévio pode ocasionar sangramentos profusos e óbito. Entretanto, as lesões específicas do segmento retro-hepático exigem manobras cirúrgicas complexas, tanto para a obtenção do controle vascular quanto para a exposição da lesão em si^7-11^.

Ao longo do tempo, duas técnicas destacaram-se para a obtenção do controle vascular das lesões da veia cava retro-hepática: a tríplice exclusão hepática (TEH), ou isolamento vascular total do fígado, descrita por Heaney em 1966, e o *shunt* átrio-caval (SAC), descrito originalmente em 1968 por Schrock^12-14^.

A vantagem do SAC consistiria na manutenção do retorno venoso pelo eixo da VCI, amenizando a redução do débito cardíaco consequente ao clampeamento acima das veias renais, que é necessário para a manobra de TEH. Por outro lado, o isolamento vascular do fígado não exige a abertura de uma câmara cardíaca, evitando uma série de complicações relacionadas ao SAC^15-17^.

A partir de 1998, relatos de técnicas endovasculares, como o implante de *stents* revestidos, e de estratégias híbridas, como a oclusão endovascular por balões para hemostasia temporária seguida da abordagem cirúrgica da lesão, tornaram-se mais frequentes na literatura. Essas técnicas representam uma abordagem menos invasiva e podem apresentar melhores desfechos. Todavia, a disponibilidade de materiais e de uma equipe treinada para esse tipo de procedimento limita sua utilização^18-22^.

Apesar de a elevada letalidade associada ao traumatismo do segmento retro-hepático da VCI ser um ponto de convergência entre os pesquisadores, a revisão da literatura não define, de forma consistente, qual estratégia oferece menor mortalidade no tratamento dessas lesões^6,11,23^.

## OBJETIVO

Definir qual técnica apresenta menor mortalidade e incidência de complicações no tratamento de lesões penetrantes do segmento retro-hepático da VCI em pacientes adultos submetidos a cirurgia de emergência.

## MÉTODOS

A revisão sistemática de literatura foi registrada na plataforma PROSPERO^24^, sob o número de registro CRD42023464133, e teve seu protocolo previamente publicado^25^. O trabalho não necessita de aprovação do comitê de ética por se tratar de uma revisão sistemática.

### Critérios de elegibilidade

#### Tipos de estudos

O processo de revisão foi orientado pelo *Cochrane Handbook for Systematic Review of Interventions* ^26^ e pelo Preferred Reporting Items for Systematic Reviews and Meta-Analyses (PRISMA) 2020. Foram considerados estudos de ensaios clínicos controlados randomizados (ECRs) nos delineamentos paralelo, em *cluster* e quase-ECRs. Estudos não randomizados de intervenções, desde que incluíssem ao menos dois grupos comparativos de interesse, também foram contemplados, assim como estudos observacionais, séries de casos e relatos de caso, quando não fossem identificados estudos ECRs ou quase-ECRs referentes ao tema.

Todos os estudos que descreveram casos de lesões penetrantes do segmento retro-hepático da VCI em pacientes adultos, tratados por TEH, SAC ou técnicas endovasculares/híbridas (E/H), submetidos a cirurgia de emergência, foram classificados.

#### Tipos de participantes

Foram incluídos pacientes de ambos os sexos, com idade igual ou superior a 18 anos, submetidos a cirurgia de emergência por lesões penetrantes do segmento retro-hepático da VCI, confirmadas por exames de imagem ou por exploração cirúrgica.

#### Tipos de intervenção

As intervenções consideradas para o tratamento de lesões penetrantes do segmento retro-hepático da VCI foram: a TEH, o implante de SAC e o uso de técnicas E/H.

### Fontes de informações

#### Método de busca para identificação dos estudos

A estratégia de busca elaborada com termos do Medical Subject Headings (((vena cava[Title/Abstract]) AND (trauma OR injuries)[Title/Abstract])) foi aplicada nas seguintes bases de dados: Literatura Latino-Americana e do Caribe em Ciências da Saúde, Web of Science, PubMed/MEDLINE, Scopus e Embase. Nenhum filtro foi aplicado durante o processo. Uma busca por revisões elaboradas sobre o mesmo tema foi feita na Cochrane Library durante a fase de planejamento do estudo, sem detecção de qualquer produção relevante. Ensaios em andamento ou não publicados também foram pesquisados nas plataformas ClinicalTrials.gov e na International Clinical Trials Registry Platform, por meio do portal da Organização Mundial de Saúde (OMS).

#### Seleção de estudos

A seleção dos estudos foi realizada em três etapas. Na primeira, o *software* Rayyan^27^ foi utilizado para gerenciar os artigos identificados nas bases de dados, classificando cada arquivo e excluindo duplicatas manualmente. Na segunda etapa, dois revisores, de forma independente, analisaram títulos e resumos dos artigos selecionados, aplicando os critérios de elegibilidade previamente definidos. A terceira etapa, realizada por outros dois revisores, consistiu na leitura independente de cada artigo incluído na fase anterior, resultando na seleção final dos estudos utilizados para a extração de dados.

O processo de seleção encontra-se detalhado no diagrama de fluxo PRISMA 2020^28^. As divergências ocorridas durante esse processo foram solucionadas por meio de discussão entre os membros elaboradores.

#### Extração e gerenciamento dos dados

O formulário de extração de dados (Tabela 1) foi executado por dois revisores de forma independente, considerando as seguintes variáveis dos artigos:

**Tabela 1:** Formulário de extração dos dados.

**Variáveis**	**Resultado**
Código do artigo:	
Autor:	
Título:	
Ano de publicação:	
País:	
Qualificação do estudo:	
Desenho do estudo:	
Método de análise:	
Revista publicação:	
Total da amostra:	
Total de cava retro-hepática:	
N° participantes acompanhamento:	
SAC:	
TEH:	
Técnicas E/H:	
Idade média:	
Idade mínima:	
Idade máxima:	
Sexo masculino:	
% masculino:	
Sexo feminino:	
% feminino:	
Média duração de acompanhamento:	
Tempo mínimo de acompanhamento:	
Tempo máximo de acompanhamento:	
Taxa de mortalidade SAC (%):	
Taxa de mortalidade TEH (%):	
Taxa de mortalidade E/H (%):	
Taxa de mortalidade geral (%):	
Hemoderivados SAC (ml):	
Hemoderivados TEH (ml):	
Hemoderivados E/H (ml):	
Hemoderivados geral (ml):	
Média de tempo UTI SAC:	
Mínimo de tempo UTI SAC:	
Máximo de tempo UTI SAC:	
Média de tempo UTI TEH:	
Mínimo de tempo UTI TEH:	
Máximo de tempo UTI TEH:	
Média de tempo UTI E/H:	
Mínimo de tempo UTI E/H:	
Máximo de tempo UTI E/H:	
Média de tempo UTI geral:	
Mínimo de tempo UTI geral:	
Máximo de tempo UTI geral:	
Pacientes hemodiálise TEH:	
Pacientes hemodiálise E/H:	
Tipo de população (trauma penetrante ou contuso):	
Risco de viés:	
Qual o risco de viés:	

SAC = *shunt* átrio-caval; TEH = tríplice exclusão hepática; E/H = endovasculares/híbridas; UTI = unidade de terapia intensiva.

- Desenho do estudo;
- Método de análise;
- Medidas de resultado;
- Duração do acompanhamento;
- Número de participantes na linha de base e acompanhamento;
- Tipo de população;
- Percentual (%) por sexo;
- Idade média (desvio-padrão [DP]);
- Covariáveis ajustadas;
- Intervenção utilizada;
- Dados para calcular as diferenças quanto aos desfechos clínicos nos resultados das intervenções: porcentagem de sobrevida, uso de hemoderivados, tempo de internação em unidade de terapia intensiva, necessidade de hemodiálise;
- Fontes de financiamento do estudo e declarações de interesses dos autores;

#### Análise estastística

As informações da caracterização amostral foram digitadas em planilha no *software* Microsoft® Office Excel® 2016. Para a estatística descritiva, foram construídos tabelas e gráficos para apresentação dos resultados.

Para a estatística analítica, utilizaram-se testes de aderência para tabelas univariadas, o teste qui-quadrado de independência para comparações bivariadas e a análise de variância de um critério para comparação da taxa de mortalidade entre as técnicas.

As análises descritiva e analítica foram realizadas no *software* BioEstat 5.4, adotando-se o nível de significância α = 0,05, ou 5%.

## RESULTADOS

A estratégia de busca identificou 12.198 estudos, dos quais 6.417 (52%) eram duplicados. Após a triagem de títulos e resumos, 5.481 artigos foram excluídos, a maioria por abordarem traumatismos de outros segmentos da VCI e não da porção retro-hepática. Após a avaliação dos textos completos, 284 artigos foram excluídos por não atenderem aos critérios de inclusão. Assim, 16 artigos foram incluídos nesta revisão. A Figura 1 retrata o fluxograma PRISMA de seleção.

**Figura 1:** Fluxograma PRISMA 2020. PRISMA = Preferred Reporting Items for Systematic reviews and Meta-Analyses. Razão 1 = artigos fora dos critérios de inclusão; Razão 2 = amostras inadequadas.

Os estudos foram publicados entre 1970 e 2021, sendo que mais de 50,0% ocorreram entre 1970 e 1999 (Tabela 2). As séries de casos representaram 81,3%, enquanto os relatos de caso corresponderam a 18,7%. A presente revisão não identificou ECRs paralelos, em *cluster* ou quase-ECRs. A Tabela 2 apresenta os artigos selecionados.

**Tabela 2:** Artigos selecionados para a revisão.

**Título**	**Autor**	**Ano de publicação**	**País**
Successful Management of an Injury to the Suprarenal Inferior Vena Cava^29^	Bricker DL, Wukasch DC.	1970	EUA
Massive Venous Injuries Associatad With Penetranting Wounds Of The Liver^30^	Burns RP, Britt LG.	1975	EUA
Traumatic injuries of the inferior vena cava^31^	Graham JM, Mattox KL, Beall AC, DeBakey ME.	1978	EUA
Inferior vena cava injuries - a continuing challenge^32^	Millikan JS, Moore EE, Cogbill TH, Kashuk JL.	1983	EUA
Abdominal Venous Injuries^33^	Wiencek RG, Wilson RF.	1986	EUA
Contemporary management strategy for major inferior vena caval injuries^34^	Klein SR, Baumgartner FJ, Bongard FS.	1994	EUA
Penetrating injuries of the abdominal inferior vena cava^35^	Deagiannis E, Velmahos G C, Levy R D, Souter I, Benn C A, Saadia R.	1996	África
The ongoing challenge of retroperitoneal vascular injuries^36^	Coimbra R, Hoyt D, Winchell R, Simons R, Fortlage D, Garcia J.	1996	EUA
Management of penetrating juxtahepatic inferior vena cava injuries under total vascular occlusion^37^	S C Khaneja, W F Pizzi, P S Barie, N Ahmed.	1997	EUA
Prognostic factors in patients with inferior vena cava injuries ^38^	Rosengart MR, Smith DR, Melton SM, May AK, Rue LW.	1999	EUA
Abdominal vena caval injuries: Outcomesremain dismal^39^	Hansen CJ, Bernadas C, West MA, Ney AL, Muehlstedt S, Cohen M, Rodriguez JL.	2000	EUA
Repair of a Juxtahepatic Inferior Vena Cava Injury Using a simple endovascular technique^40^	Angeles AP, Agarwal N, Lynd C Jr.	2004	EUA
Shunt atriocava. Report of two cases^41^	Soto S, Oettinger R.	2005	Chile
Traumatismos de veia cava inferior^7^	Costa CA, Baptista-Silva JCC, Rodrigues LME, Mendonça FLP, Paiva TS, Burihan E.	2005	Brasil
Outcome of ligation of the inferior vena cava in the morden era^42^	Sullivan PS, Dente CJ, Patel S, Carmichael M, Srinivasan JK, Wyrzykowski AD, Nicholas JM, Salomone JP, Ingram WL, Vercruysse GA, Rozycki GS, Feliciano DV.	2010	EUA
Lessons learned from treating 114 inferior vena cava injuries at a limited resources environment^6^	Góes Junior AMO, Silva KTB, Furlaneto IP, Abib SCV.	2021	Brasil

Os artigos selecionados permitiram reunir 921 casos de lesões de VCI, dos quais apenas 206 (22,4%) corresponderam ao segmento retro-hepático. Entre estes, 195 pacientes (21,2%) tiveram o curso de acompanhamento descrito.

Pacientes do sexo masculino representaram 90,3% da população estudada, enquanto o sexo feminino correspondeu a 9,7%, com idade média de 27,3 anos.

Foi possível identificar a técnica utilizada para tratamento em 96 casos. Não houve diferença estatisticamente significativa quanto à frequência com que a TEH (49 casos) e o SAC (38 casos) foram executados. Entretanto, as técnicas E/H foram menos frequentes (nove casos) (p < 0,0001).

A mortalidade global dos casos de lesões da cava retro-hepática foi de 53,8%. Casos tratados com TEH apresentaram mortalidade de 43,0%; nos casos em que SAC foi implantado, a mortalidade foi de 46,4%; e técnicas H/E estiveram associadas a 34,0% de mortalidade. Entretanto, a comparação entre as taxas de mortalidade das três técnicas não demonstrou diferença estatisticamente significativa (p = 0,9085). A Tabela 3 apresenta os dados estatísticos da comparação de mortalidade.

**Tabela 3:** Análise estatística do tipo ANOVA para comparação da mortalidade geral e entre as técnicas.

**Variáveis**	**Número de casos**	**Taxa de mortalidade**		**Valor de p** *
		**Variação**	**Média**	
Mortalidade geral	96	0-93%	53,8%	
Técnica SAC	38	0-100%	46,4%	0,9085
Técnica TEH	49	0-80%	43,0%	
Técnica E/H	9	0-68%	34,0%	
SAC x TEH	----	----	----	0,2723
SAC x E/H	----	----	----	0,2351
TEH x E/H	----	----	----	0,3706
Valor de p	< 0,0001	----	-----	----

SAC = *shunt* átrio-caval; TEH = tríplice exclusão hepática; E/H: endovascular/híbrida; ANOVA = análise de variância (*analysis of variance*).

* Valor de p 0.

Quanto às variáveis para comparação do desfecho clínico, informações sobre o uso de hemoderivados, tempo de internação em unidade de terapia intensiva e necessidade de hemodiálise não foram identificadas na maioria dos artigos, o que inviabilizou a análise dessas variáveis.

## DISCUSSÃO

Melhorias no atendimento pré-hospitalar e a redução no tempo de transporte permitiram que pacientes gravemente traumatizados, que antes evoluíam para óbito na cena, passassem a chegar com vida aos hospitais. Esses casos exigem abordagens rápidas e eficientes^30,34,43-45^.

A elevada mortalidade das lesões retro-hepáticas da VCI está relacionada às dificuldades técnicas de acesso cirúrgico, à velocidade da perda volêmica, ao tamanho da lesão e ao tempo decorrido até a obtenção da hemostasia^35,38-40,46,47^. A taxa de mortalidade geral encontrada neste estudo foi superior a 50%, em consonância com a literatura sobre o tema, enfatizando o desafio que esse tipo de trauma representa^31,33,42^.

A literatura apresenta dados controversos sobre o SAC, descrito por Schrock em 1968. Além da alta mortalidade, requer manobras cirúrgicas complexas, como a introdução do *shunt* através de uma abertura no átrio direito, com sua sutura em bolsa. Além das dificuldades técnicas de realização, estão descritas a ocorrência de embolia gasosa e de lesões cardíacas e vasculares durante a inserção do *shunt* ^32,48-50^. Entretanto, também há relatos exitosos, e os adeptos da técnica alegam que a manutenção do retorno venoso pelo eixo da cava inferior, enquanto se procede ao reparo da lesão, reduziria as repercussões hemodinâmicas da hipovolemia, oferecendo uma vantagem em relação à oclusão da veia cava, necessariamente realizada durante a TEH^41^.

A realização da TEH, por sua vez, também está associada a alta mortalidade. Contudo, a ausência de diferença estatisticamente significativa em relação àquela observada com o SAC sugere que a letalidade não está associada à estratégia empregada para o tratamento (p = 0,2723).

Embora a TEH possa ser obtida pelo clampeamento da veia cava entre o fígado e o diafragma, essa opção deve ficar restrita a procedimentos eletivos, como o transplante hepático^12^, pois o espaço exíguo aumenta o risco de iatrogenias. No contexto do trauma, quando o acesso precisa ser rápido, a forma mais segura de obter o controle da cava inferior acima do fígado é em sua porção intrapericárdica, que pode ser exposta tanto por esternotomia quanto por toracotomia anterolateral direita^51^, sendo esta última preferida pelos autores, já que a comunicação dos acessos (torácico e abdominal), inclusive com a possibilidade de toracofrenolaparotomia, permite ampla mobilização hepática, favorecendo a exposição da lesão vascular^52^.

A diminuição do retorno venoso provocada pela oclusão da cava inferior pode reduzir a pressão arterial. Contudo, o clampeamento da aorta descendente por toracotomia anterolateral esquerda ou da aorta abdominal em seu segmento supracelíaco pode compensar parcialmente os efeitos hemodinâmicos da oclusão venosa em pacientes com pressão arterial sistólica inferior a 90 mmHg^37^.

As séries de casos encontradas durante esta revisão sistemática demonstraram mortalidade entre 30 e 55% em pacientes tratados com TEH ^35,36^, e entre 68 e 100%^29,41,42^ quando o SAC foi utilizado. Entretanto, como não foi detectada diferença estatisticamente significativa quanto à mortalidade entre essas duas estratégias, cabe ao cirurgião definir qual técnica utilizar, considerando seu treinamento, experiência e disponibilidade de recursos.

O reduzido número de casos em que foram utilizadas técnicas E/H já era esperado, não apenas por se tratar de opções mais recentemente desenvolvidas, mas também pela escassez de recursos técnicos e materiais necessários para sua implementação. Devido ao menor quantitativo, nesta pesquisa, os casos tratados exclusivamente com técnicas endovasculares e aqueles em que foram empregadas estratégias híbridas foram analisados como um único grupo.

Embora a técnica de Resuscitative Endovascular Balloon Occlusion of the Aorta tenha sido originalmente descrita para oclusão aórtica com balão endovascular, essa passou a ser utilizada também no tratamento de lesões de VCI, sendo inclusive cunhado o termo Resuscitative Endovascular Balloon Occlusion of the Vena Cava. A partir dos anos 2000, tornou-se cada vez mais frequente a descrição de *stents* revestidos em traumatismos da veia cava^53-57^.

As técnicas exclusivamente endovasculares, como o implante de *stents* revestidos, e as estratégias híbridas oferecem alternativas minimamente invasivas e eficazes para um traumatismo complexo^58-60^. De acordo com esta revisão da literatura, apresentam taxas de mortalidade relativamente baixas e, quando disponíveis, devem certamente ser consideradas.

As estratégias híbridas consistem em obter a oclusão vascular pela insuflação de balões intraluminais, inseridos por via percutânea ou por exposição cirúrgica dos vasos. Essa abordagem pode promover hemostasia temporária, proporcionando um campo cirúrgico relativamente exangue, favorecendo reparos vasculares com menor risco de estenoses significativas e reduzindo a chance de oclusão de tributárias da veia cava^61-63^.

Embora a mortalidade associada à TEH, ao SAC e às técnicas E/H não tenha sido estatisticamente diferente nesta pesquisa, esse resultado pode estar relacionado ao reduzido tamanho da amostra e à heterogeneidade dos grupos comparados.

Entre as limitações desta revisão, destaca-se o fato de que, devido à não identificação de estudos de maior qualidade, apenas relatos de caso e séries de casos foram incluídos, os quais apresentaram grande heterogeneidade na descrição das variáveis. Em todos os estudos havia lesões associadas a outras estruturas anatômicas, e nenhum deles foi desenhado com o objetivo de comparar o desfecho das três intervenções analisadas. Além disso, entre as quatro variáveis propostas em nosso protocolo^25^ para avaliar os desfechos clínicos das intervenções (porcentagem de sobrevida, uso de hemoderivados, tempo de internação em unidade de terapia intensiva e necessidade de hemodiálise), apenas a sobrevida dos pacientes foi descrita em todos os artigos selecionados, gerando um viés que prejudicou a análise estatística e impactou negativamente o nível de evidência. Além disso, em todos os casos incluídos havia outras lesões associadas, o que impede afirmar que os desfechos possam ser atribuídos especificamente à lesão do segmento retro-hepático da VCI, acarretando vieses por carência de dados e fatores de confusão.

O protocolo desta revisão sistemática^25^ contemplava a aplicação do instrumento ROBINS-I para avaliação do risco de viés nos estudos selecionados, uma vez que a pesquisa preliminar havia identificado apenas estudos não randomizados sobre o tema. Entretanto, a ausência de estudos de coorte e de casos-controle comparando os três tipos de intervenção investigados, somada às limitações dos estudos incluídos, não permitiu a aplicação do instrumento proposto em nosso protocolo inicial.

A realização de trabalhos multicêntricos prospectivos, com padronização da coleta de informações sobre a evolução e o desfecho dos casos, bem como da técnica empregada, poderia contribuir para reduzir tais limitações e oferecer conclusões mais precisas. Entretanto, a gravidade do tipo de lesão estudada dificulta a homogeneidade das condutas, uma vez que a expertise e os materiais necessários não estão uniformemente disponíveis, tornando improvável a implementação de estudos dessa natureza.

## CONCLUSÃO

A revisão sobre os desfechos de casos de lesões penetrantes do segmento retrohepático da VCI em pacientes adultos operados em caráter de emergência, tratados com a utilização do SAC, da TEH ou de técnicas E/H, não demostrou superioridade ou inferioridade de nenhuma das técnicas quanto à mortalidade.

## Data Availability

The data on which this study is based are available on request from the corresponding author, AMOG (goesjunior@ufpa.br).
